# A Phase 2 Study to Assess the Immunomodulatory Capacity of a Lecithin-based Delivery System of Curcumin in Endometrial Cancer

**DOI:** 10.3389/fnut.2018.00138

**Published:** 2019-01-11

**Authors:** Sandra Tuyaerts, Klara Rombauts, Tina Everaert, An M. T. Van Nuffel, Frédéric Amant

**Affiliations:** ^1^Laboratory of Gynaecologic Oncology, Department of Oncology, KU Leuven, Leuven, Belgium; ^2^Leuven Cancer Institute, Leuven, Belgium; ^3^Anticancer Fund, Strombeek-Bever, Belgium; ^4^Center for Gynecologic Oncology Amsterdam, Amsterdam University Medical Center, Antoni van Leeuwenhoek Hospital, Netherlands Cancer Institute, Amsterdam, Netherlands

**Keywords:** curcumin, immunomodulation, endometrial cancer, inflammatory biomarkers, quality of life

## Abstract

Curcumin is a botanical with anti-tumor and immunomodulatory properties. We hypothesized that curcumin supplementation might influence inflammatory biomarker levels in endometrial carcinoma (EC). In this open-label, non-randomized phase 2 study (NCT02017353), seven EC patients consumed 2 g/day Curcumin Phytosome (CP) orally for 2 weeks. Blood was taken at baseline, days 1, 7, 14, and 21. The following analytes were measured: curcuminoids and metabolites, 56 inflammatory biomarkers, COX-2, frequencies of myeloid-derived suppressor cells, dendritic cells and NK cells, expression of MHC molecules on leukocytes and monocytes and activation/memory status of T cells. Patients completed quality of life (QoL) questionnaires at baseline and end of treatment. Curcumin metabolites were detectable in plasma upon CP intake. CP downregulated MHC expression levels on leukocytes (*P* = 0.0313), the frequency of monocytes (*P* = 0.0114) and ICOS expression by CD8^+^ T cells (*P* = 0.0002). However, CP upregulated CD69 levels on CD16^−^ NK cells (*P* = 0.0313). No differences were observed regarding inflammatory biomarkers, frequencies of other immune cell types, T cell activation and COX-2 expression. A non-significant trend to improved QoL was observed. Overall, CP-induced immunomodulatory effects in EC were modest without significant QoL changes. Given the small population and the observed variability in inter-patient biomarker levels, more research is necessary to explore whether benefits of CP can be obtained in EC by different supplementation regimens.

**Clinical Trial Registration:** www.ClinicalTrials.gov, identifier NCT02017353; www.clinicaltrialsregister.eu, identifier 2013-001737-40.

## Introduction

Curcumin is a polyphenol derived from the plant *Curcuma longa (common name Turmeric)*. It is used in traditional Ayurvedic medicine. Besides curcumin, turmeric also contains demethoxycurcumin and bisdemethoxycurcumin, together forming the curcuminoids (1). Several preclinical studies documented the anticancer effects of curcumin, by modulating molecules implicated in cancer, such as NF-κB, COX-2, lipooxygenase, and protein kinase C (2, 3). Furthermore, curcumin has also been shown to potentiate the anticancer effects of conventional anticancer therapies such as chemotherapy or radiation by sensitizing cancer cells to their cytocidal effects (4, 5).

Besides its direct effects on cancer cells, emerging data point toward anti-inflammatory and immune-modulatory effects of curcumin that could play a role in its anti-tumor effects (6). Curcumin has been shown to inhibit the accumulation of myeloid-derived suppressor cells (MDSC) and their interaction with cancer cells and induces the differentiation/maturation of MDSC (7). Curcumin reduced intratumoral IL-6 production and metastasis formation in a breast cancer model and, when combined with cryoablation, induced robust anti-tumor T cell immunity and reduced tumor growth (8). In RAW 264.7 murine macrophages, a curcumin formulation significantly decreased the LPS-induced pro-inflammatory mediators NO, PGE_2_, and IL-6 by inhibiting activation of NF-κB (9). In IFNγ-stimulated murine bone marrow-derived dendritic cells (DCs), curcumin has shown to inhibit the expression and functionality of indoleamine-2,3-dioxygenase, a major immunosuppressive enzyme in tumor immunology (10). Through inhibition of COX-2, curcumin also reduced PGE_2_ production, which exerts potent immunosuppressive effects in the tumor microenvironment (1). Recently, curcumin has shown to inhibit inflammation-mediated PD-L1 expression, an immune checkpoint enabling tumors to evade the immune response (11). In contrast, several features that could be detrimental to anti-tumor immunity have also been attributed to curcumin, such as induction of IL-10 (12), inhibition of T cell responses (13), inhibition of dendritic cell maturation (13), and induction of regulatory T cells (14).

A major obstacle hampering the implementation of curcumin in the clinic is its poor bioavailability. Most preclinical studies have investigated the effects of curcumin at dosages impossible to obtain after oral intake of curcumin. Various approaches have been developed to improve the bioavailability of curcumin. A first approach is the use of the adjuvant piperine, which increases curcumin bioavailability by inhibiting the enzymes UDP-glucuronyltransferase (UGT) and sulfotransfereases (SULT) that are responsible for transformation of curcumin into curcumin glucuronide and curcumin sulfate (15–17). Second, various formulations of curcumin delivery systems have been developed to improve bioavailability. This comprises the use of nanoparticles/nanoemulsions, complexes with phospholipids, formulation with soluble dietary fibers, micronization, micellization, and other agents (17–22). Finally, curcumin derivatives and analogs have been synthesized to improve the biological activity of curcumin. However, although many curcumin analogs have shown improved biological activity over curcumin, specific evaluations of structural analogs and/or derivatives of curcumin to improved tissue and plasma distribution are lacking (17). Although all these formulations claim to improve curcumin bioavailability, plasma levels remain quite low, due to rapid metabolism and possibly uptake into tissues. Moreover, extensive variability in the studies makes it difficult to directly compare and conclude which formulation is better than the other. Curcumin Phytosome (CP) is a patented formulation of turmeric extract with soy lecithin. These two components form a non-covalent adduct in a 1:2 ratio, and two parts of microcrystalline cellulose are added to improve formulation, with an overall curcuminoid content of 20% (15). This formulation improves the plasma levels of curcumin and its metabolites (23) and is documented with preclinical and clinical pharmacokinetic studies (23, 24), supported by GLP preclinical safety studies (personal communication with Indena S.p.A., Investigator's Brochure) and has been used in a number of clinical studies (25–28). Another drawback of curcumin is its potential to interfere with several assays (pan-assay interference compounds or PAINS), which might result in overestimation of its biological activities (29).

Curcumin-containing dietary supplements have been used in various clinical trials in cancer or other diseases without major side effects and are generally regarded as safe (GRAS) by the US Food and Drug Administration (FDA). In this phase 2 study, we evaluated the effects of a daily intake of 2 g CP by EC patients during a 2-week, oncological treatment-free interval. The objectives of the study included evaluation of the immunomodulatory effects of CP, bioavailability and impact of the treatment on patient's quality of life.

## Materials and Methods

### Patient Recruitment and Treatment

The trial was approved by the local ethics committee of the University Hospital Leuven (S55201) and by the Federal Agency for Medicines and Health Products (FAMHP; EudraCT: 2013-001737-40). Patients with histologically confirmed EC and no life-threatening metastases were recruited by the department of gynecological oncology of UZ Leuven. Exclusion criteria were: other active malignancy, documented autoimmune disease or immune deficiency, ongoing immunosuppressive therapy and current enrollment in other clinical trials. All patients were asked to complete a questionnaire concerning the QoL before and after CP supplementation. Each patient had to document their daily consumption of certain foods or food supplements specified in a dietary list. Written informed consent was obtained from each patient before enrollment.

Curcumin Phytosome (Meriva®, CP) was provided by Indena SpA and manufactured into capsules (Curcuphyt®) by nutrisan nv. The capsules contained 500 mg of CP, corresponding to 100 mg of curcuminoids. Patients were supplemented for 2 weeks with 2 g CP per day in a time period during which they did not receive any oncological treatment.

### Blood and Urine Collection

Blood samples were collected at baseline, on the first day of curcumin intake, then once weekly during the supplementation period, and finally 1 week after the end of supplementation. On each day of blood collection, patients were requested to take their noon intake of curcumin in the hospital and blood samples were collected at different time points following curcumin intake (15-30-60-120 min). Blood was collected in one EDTA and one heparin tube for the measurement of hemoglobin, red blood cells, white blood cells, thrombocytes, CA125 and CRP at the central laboratory. In addition, blood was collected in 1 supplementary EDTA tube and 4 supplementary heparin tubes and transferred to the laboratory of gynecological oncology for separation of plasma and cellular fraction. Indomethacin was added to the EDTA tube before centrifugation. Plasma was aliquoted and stored at −80°C. On the first day of curcumin intake, patients were requested to perform a 24-h urine collection. The collected urine was centrifuged, aliquoted, and stored at −80°C.

### Measurement of Plasma Curcumin Levels

For extraction, 500 μL of acetone/0.25 M formic acid (9:1, *v/v*) was added to 250 μL of plasma sample. After vortexing, the mixture was kept at −20°C for 30 min and subsequently centrifuged at 16,100 × g for 20 min at 4°C. The supernatant was evaporated to dryness overnight using a Savant DNA speed vac DNA120 centrifugal evaporator. The dried residue was resuspended in 50 μL of 0.1% acetic acid/acetonitrile (0.1% acetic acid) (40:60, *v/v*), centrifuged at 16,100 × g for 3 min and the volume injected on to the column was 20 μL in duplicate. Curcuminoids were separated and quantified using a Waters Alliance 2695 separations module with a 100 μL injection loop and Waters 2487 UV detector, with a HyPurity C18 (2.1 × 150 mm, 3 μm) column connected to a HyPurity C18 (2.1 × 10 mm, 3 μm) guard cartridge plus a KrudKatcher (5 μm) disposable pre-column filter. The samples were analyzed in negative electrospray ionization (ESI) mode. The data was acquired using MassLynx software v4.0. A single injection for each sample was performed. The calibration lines were constructed using pure standards for curcumin, curcumin glucuronide and curcumin sulfate by injection of a 10 μL aliquot for each standard onto the liquid chromatography/electrospray ionization mass spectrometry (LC-ESI-MS/MS). For desmethoxycurcumin, a standard was not available and levels were estimated using the curcumin calibration line.

### Quality of Life Assessment

QoL scores were assessed using the EORTC QLQ-C30 version 3.0 and EQ-5D questionnaires. Patients were asked to complete the questionnaires at baseline and at the last day of curcumin intake. QoL scores are presented as means ± standard deviations.

For the EORTC QLQ-C30 questionnaire, five functional scores (emotional, role, cognitive, physical, and social) were pooled and a summary score was calculated according to Giesinger et al (30) using SPSS software. A higher score indicates a better health for functioning and global health status, whereas for the symptom scales a lower score indicates a lower level of symptom burden.

The EQ-5D questionnaire consists of 2 parts—the EQ-5D descriptive system and the EQ visual analog scale (EQ VAS). The EQ-5D-3L descriptive system comprises the following 5 dimensions: mobility, self-care, usual activities, pain/discomfort, and anxiety/depression. EQ-5D health states, defined by the EQ-5D descriptive system, were converted into a single summary index according to the EQ-5D user guide. The EQ VAS records the respondent's self-rated health on a vertical, analog scale where the endpoints are labeled “Best imaginable health state” and “Worst imaginable health state.” For both the EQ-5D index and EQ VAS, a higher score indicates a better health status.

### Peripheral Blood Mononuclear Cells (PBMC) Isolation

Peripheral blood from 4 heparin tubes was diluted 1:2 in PBS and PBMC were isolated using Lymphoprep™ (AXIS-SHIELD) density gradient centrifugation and counted with Türck's solution. PBMC were cryopreserved in 90% human AB serum (Sera Laboratories International) with 10% DMSO at 5–10 × 10^6^ cells per vial using CoolCell freezing containers (BioCision), and stored in liquid nitrogen until further use.

### Measurement of Soluble Analytes

PGE_2_ was measured from indomethacin-treated EDTA plasma using the competitive Biotrak™ enzymeimmunoassay system (GE Healthcare). Neopterin levels were measured from heparin plasma using an enzyme-linked competitive immunosorbent assay (Neopterin ELISA; Immuno Biological Laboratories). Measurement of HMGB1 was performed using the HMGB1 ELISA kit from (IBL). Lactate was measured in heparin plasma by use of the L-Lactate assay kit colorimetric (Abcam). A deproteinization step was performed on the samples prior to the assay by adding trichloroacetic acid. For all assays, optical density was read at 450 nm using the Multiscan FC reader and ScanIt software (Thermo Scientific).

Luminex assays were performed on heparin plasma samples: custom ordered kit (EMD Millipore, HCCBP1MAG-58K) to analyze CA15-3, CEA, Leptin, MIF and Prolactin and Procartaplex Immunoassay Kit (Affymetrix-eBioscience) to analyze BDNF, Eotaxin/CCL11, EGF, FGF-2, GM-CSF, GROα/CXCL1, HGF, NGFβ, LIF, IFNα, IFNγ, IL-1β, IL-1α, IL-1RA, IL-2, IL-4, IL-5, IL-6, IL-7, IL-8/CXCL8, IL-9, IL-10, IL-12 p70, IL-13, IL-15, IL-17A, IL-18, IL-21, IL-22, IL-23, IL-27, IL-31, IP-10/CXCL10, MCP-1/CCL2, MIP-1α/CCL3, MIP-1β/CCL4, RANTES/CCL5, SDF-1α/CXCL12, TNFα, TNFβ/LTA, PDGF-BB, PlGF, SCF, VEGF-A, VEGF-D. Plates were read on a Luminex 200 system (Bio-Rad Laboratories). Data were analyzed using Bio-Plex Manager software (Bio-Rad Laboratories).

### Flow Cytometric Analyses

Cryopreserved PBMC from each time point of every patient were thawed and counted using trypan blue (Sigma-Aldrich).

For analysis of COX-2 expression, 1.5 × 10^6^ cells were plated per 24-well (2 wells for each time point) of a low-adherence 24-well plate. One well was stimulated with 1 μg/mL LPS (K12, Invivogen) and the other was left untreated. Cells were incubated at 37°C and 5% CO_2_ for 5 h, after which they were harvested. To exclude culture-induced effects, COX-2 expression was also analyzed on freshly thawed PBMC. Cells were stained with a fixable viability dye (Fixable Viability Dye eFluor 506, eBioscience) followed by Fc receptor blocking with 10% normal goat serum (Sigma-Aldrich). Next, the following antibodies were added: CD3-FITC (eBioscience), CD14 PerCP-Cy5.5 (BD Pharmingen), CD56-PE-Cy7 (BioLegend) and CD19-eFluor450 (eBioscience) for 30 min at 4°C. After washing, samples were fixed using fixation/permeabilization buffer (BD Biosciences Cytofix Cytoperm kit) for 20 min at 4°C. Cells were subsequently incubated with either no antibody (unstained control), mouse IgG1-PE isotype control antibody (BD Biosciences) or mouse anti-Human COX-2-PE antibody (BD Biosciences).

For PBMC phenotyping, cell suspensions were stained with a fixable viability dye, followed by Fc receptor blocking. The antibody staining panels used to identify the different cellular populations in this study are described in Table 1. For Treg analysis, the samples were fixed and permeabilized using the FoxP3 Staining Buffer Set (eBioscience) and stained with FoxP3-APC (eBioscience). For CD247 staining, samples were fixed with PBS/0.5% paraformaldehyde for 20 min at room temperature (RT) in the dark. After 2 washing steps with PBS/Tween, 100 μL cold digitonin solution (10 μg/mL in PBS) was added, followed by either mouse IgG1-PE isotype (FMO; BD Biosciences) or mouse anti-human CD247 antibody (Beckman Coulter) for 30 min at room temperature.

**Table 1:** Membrane antigen flow cytometry staining panels.

**PANEL MEMBRANE ANTIGENS**									
**1**	**2**	**3**	**4**	**5**	**6**	**7**	**8**	**9**	**10** **+** **11**
/	Mouse IgG1 FITC BD Pharmingen Clone MOPC-21 (RUO)	CD4-FITC BDPharmingen Clone RPA-T4	/	CD8-FITC Biolegend Clone HIT8a	HLA-ABC-FITC BioLegend Clone W6/32	CD3-FITC eBioscience Clone SK7	HLA-ABC-FITC BioLegend Clone W6/32	CD4-FITC BD Pharmingen Clone RPA T4	CD3-FITC eBioscience Clone SK7
/	Mouse IgG1 PE BD Pharmingen Clone MOPC-31C	Tim3-PE BioLegend Clone F38-2E2	CD161-PE Biolegend Clone HP-3G10	CD137-PE BD Pharmingen Clone 4B4-1	HLA-DR-PE BioLegend Clone L243	CCR7-PE eBioscience Clone 3D12	HLA-E-PE BioLegend Clone 3D12	CD25-PE BD Pharmingen Clone PC61 (RUO)	/
/	Mouse IgG1 PerCp-Cy5.5 BD Pharmingen Clone MOPC-21 (RUO)	ICOS-PerCp-Cy5.5 BioLegend Clone C398.4A	CD16-PerCp-Cy5.5 BDPharmingen Clone 3G8	CD4-PerCp-Cy5.5 Biolegend Clone RPA-T4	CD14-PerCp-Cy5.5 BD Pharmingen Clone M5E2	CD62L-PerCp-eFluor710 eBioscience Clone DREG-56	CD14-PerCp-Cy5.5 BD Pharmingen CloneM5E2	CD127-PerCp-Cy5.5 BD Pharmingen Clone HIL-7R-M	CD16-PerCp-Cy5.5 BD Pharmingen Clone 3G8
/	Mouse IgG1 PE-Cy7 BD Pharmingen Clone G18-145	CD3-PE-Cy7 BioLegend Clone UCHT1	CD56-PE-Cy7 BioLegend Clone MEM-8	CD3-PE-Cy7 Biolegend Clone HIT3a	CD123-PE-Cy7 eBioscience Clone 6H6	CD45RO-PE-Cy7 eBioscience Clone UCHL1	/	CD3-PE-Cy7 Biolegend Clone HIT3a	CD56-PE-Cy7 Biolegend Clone MEM-8
/	Mouse IgG1 APC BD Pharmingen Clone A85-1 (RUO)	CTLA4-APC BioLegend Clone L3D10	CD69-APC eBioscience Clone FN50	/	CD11c-APC BD Pharmingen Clone HL3	CD45RA-APC eBioscience Clone HI100	HLA-G-APC BioLegend Clone 87G	/	/
/	Mouse IgG1 APC-H7 BD Pharmingen Clone X40 (RUO)	CD8-APC-H7 BD Pharmingen Clone SK1	HLA-DR-APC-H7 BD Pharmingen Clone L243	HLA-DR-APC-H7 BD Pharmingen Clone L243	CD45-APC-H7 BD Biosciences Clone 2D1	CD8-APC-H7 BD Pharmingen Clone SK1	HLA-DR-APC-H7 BD Pharmingen Clone L243	CD8-APC-H7 BD Pharmingen Clone SK1	CD8-APC-H7 BD Pharmingen Clone SK1
/	Mouse IgG1 Pacific Blue BioLegend Clone MOPC-21	PD1-Pacific Blue BioLegend Clone EH12.2H7	CD54-Pacific Blue Biolegend Clone HCD54	CD69-BV421 BD Biosciences Clone FN50	CD54-Pacific Blue Biolegend Clone HCD54	CD4-eFluor450 eBioscience Clone SK3	CD45-Pacific Blue BD Pharmingen Clone 30-F11	CD45-Pacific Blue Biolegend Clone 30-F11	CD4-eFluor450 eBioscience Clone SK3

*This table summarizes the antibodies used for membrane staining in the different panels. Panel 1, viability only; Panel 2, isotype controls; Panel 3, T cell activation markers 1; Panel 4, NK cells; Panel 5, T cell activation markers 2; Panel 6, DC; Panel 7, T cell memory; Panel 8, HLA; Panel 9, Treg; Panel 10 and 11, T cell CD247 index*.

MDSC analysis was performed on fresh whole blood instead of cryopreserved PBMC. Blood was aliquoted at 350 μL per tube and 35 μL normal goat serum was added. For MDSC enumeration, the following antibody cocktail was added: CD45-FITC (BioLegend), CD11b-PE (BioLegend), CD14-PerCP-Cy5.5 (BD Pharmingen), CD3-PE-Cy7 (BioLegend), CD19-PE-Cy7 (BioLegend), CD56-PE-Cy7 (BioLegend), CD15-APC (BioLegend), HLA-DR-APC-H7 (BD Pharmingen), and CD33-V450 (BD Horizon). After 30 min at 4°C, red blood cell lysis was performed by adding 1x Pharm Lyse (BD Biosciences). After 15 min incubation at room temperature in the dark and washing, the cells were stained with viability dye. Analysis of arginase-1 expression by MDSC was done by replacing CD45-FITC with Arginase-1-fluorescein (R&D Systems) in the abovementioned MDSC cocktail. For assessment of arginase-1 expression, cells were first stained for membrane markers as described above, subsequently fixed and permeabilized using the FoxP3 Staining Buffer Set (eBioscience) and stained with arginase-1-fluorescein (R&D Systems).

Acquisition was performed with a FACSCanto^TM^ II using BD FACSDiva^TM^ software. For all samples, between 2.5 × 10^4^ and 1 × 10^5^ cells were acquired in the live gate per sample. Data analysis was done using BD FACSDiva^TM^ software. MDSC were gated as follows: first, we gated out dead cells and debris and subsequently we gated on CD45^+^ Lin(CD3-CD19-CD56)^−^ HLA-DR^lo^ cells. Within this gate, two major MDSC subtypes were identified as CD11b^+^ CD14^−^ granulocytic MDSC and CD11b^+^ CD14^+^ monocytic MDSC. For the granulocytic MDSC, we next distinguished CD15^+^ and CD33^+^ subtypes, while monocytic MDSC are CD15^−^ and CD33^+^. Dendritic cell (DC) gating strategy was: after dead cell exclusion, cells were gated upon their CD45^+^ and CD14^−^ characteristics. On this gate CD11c^+^ CD123^−^ cells are identified as mDC and CD11c^−^ CD123^+^ are pDC. On these subsets, we assessed the expression of HLA-ABC, HLA-DR and CD54. NK cells were identified as CD56^+^ CD16^−^ or CD56^+^ CD16^+^ cells on which the expression of CD161, CD69, and HLA-DR was evaluated. T cell subsets were defined as CD3^+^, CD3^+^ CD4^+^ CD8^−^, CD3^+^ CD4^−^ CD8^+^ or CD3^+^ CD4^+^ CD8^−^CD25^+^ FoxP3^+^ CD127^lo^. On CD4^+^ and CD8^+^ T cells, we subsequently determined expression of CD69, CD137, HLA-DR, ICOS, CTLA-4, PD-1 and Tim-3. The memory phenotype of CD4^+^ and CD8^+^ T cells was determined as follows: T_naï*ve*_ (CD45RA^+^ CD45RO CD62L^+^ CCR7^+^), T_CM_ (CD45RA^−^ CD45RO^+^ CD62L^+^ CCR7^+^), T_EM_ (CD45RA^−^ CD45RO^+^ CD62L^−^ CCR7^−^) and T_EMRA_ (CD45RA^+^ CD45RO^−^ CD62L^−^ CCR7^−^). TCRζ expression was measured on CD3^+^, CD4^+^, CD8^+^ T cells, as well as in CD56^+^ and CD16^+^ NK cells.

### Statistical Analysis

For QoL scores and plasma curcumin levels, we used the nonparametric Wilcoxon matched-pairs signed rank test because of the small sample size. Concentrations of analytes were presented as mean values ± SD. Data were analyzed and, when appropriate, significance of the differences between mean values at baseline in comparison with day 14 values was determined by Wilcoxon matched-pairs test except for CA125 and CRP where it was determined by Mann-Whitney *U*-test. Differences were assumed to be significant at *P* < 0.05. One-way repeated measure ANOVA was used to test the effect of curcumin in patients at all time points. All experiments were performed in duplicates. Prism 5 software (GraphPad Software Inc.) was used to perform all statistical analyses and to generate graphs.

## Results and Discussion

### Patient Characteristics

**Figure 1:**
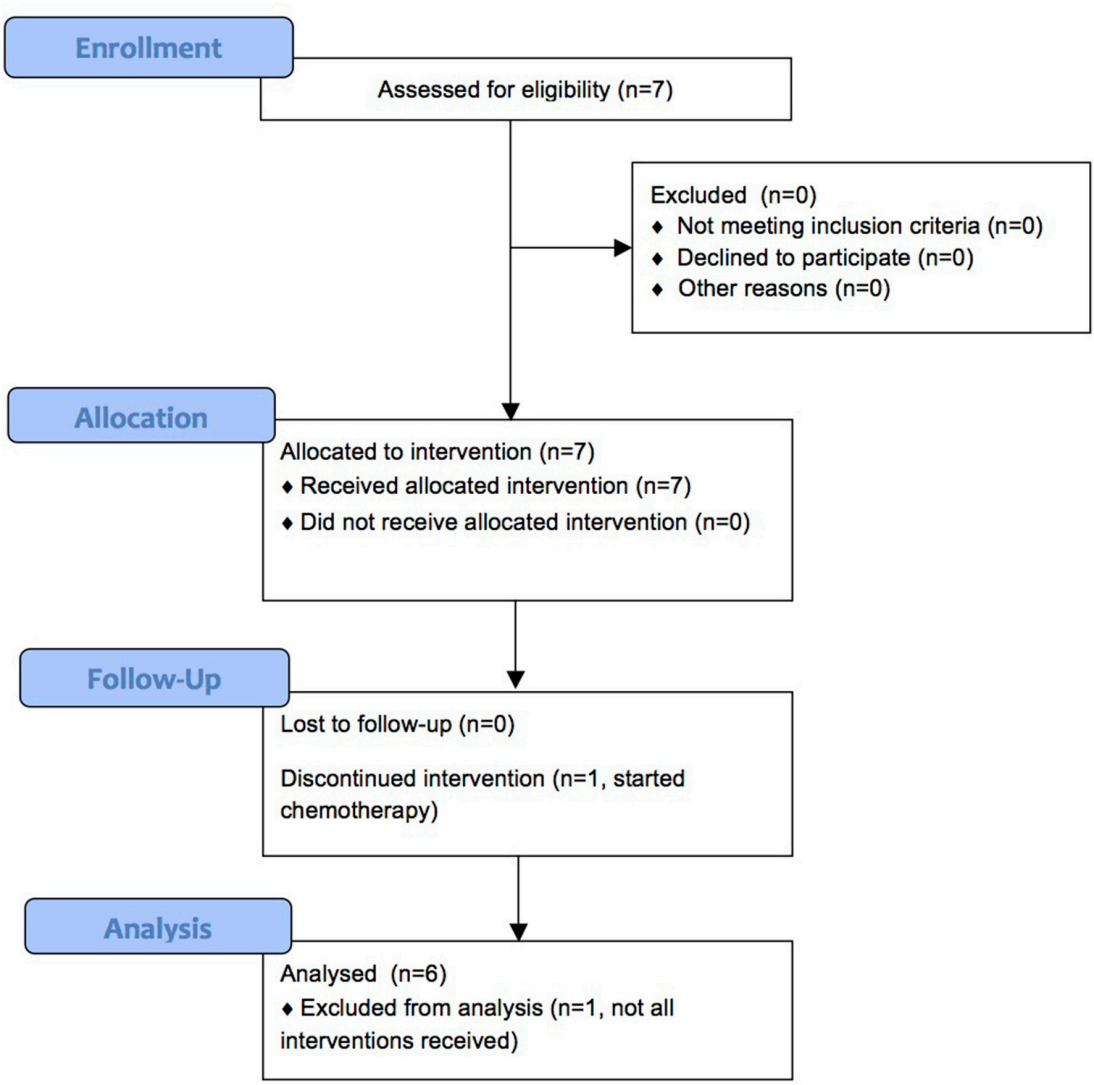
CONSORT flow diagram.

We enrolled 7 patients between September 2013 and August 2015, of which 6 completed treatment. Patient characteristics are shown in Table 2 and Figure 1 shows the CONSORT flow diagram to illustrate the progress of patients through the trial. Supplementation was administered during an oncological treatment-free interval to avoid immunomodulatory effects from standard oncological treatments. All patients had recurrent disease. In our study, we could not assess the clinical response of the patients, since they received various oncological treatments after the 2-week CP supplementation period.

**Table 2:** Patient characteristics.

**Total number of patients enrolled**	**7**
Number of evaluable patients	6
Median age (years)	77
**TUMOR TYPE**	
Endometrioid	3
Serous	2
Clear cell	1
Mesonephric	1
**FIGO STAGE**	
I	4
II	0
III	2
IV	1
**HISTOLOGICAL GRADE**	
1	2
2	2
3	3
**PRIOR TREATMENTS**	
Surgery	6
Chemotherapy	1
Radiotherapy	1
Hormonal therapy	2

All patients documented their daily consumption of certain foods or food supplements described in a dietary list. The most frequently consumed foods from the list were mushrooms, berries, broccoli, sprouts, watercress, and horseradish. Only one patient consumed a food supplement containing propolis on a daily basis which could have immunological effects (31, 32).

### Plasma Levels of Curcuminoids and Soluble Inflammatory Mediators

**Figure 2:**
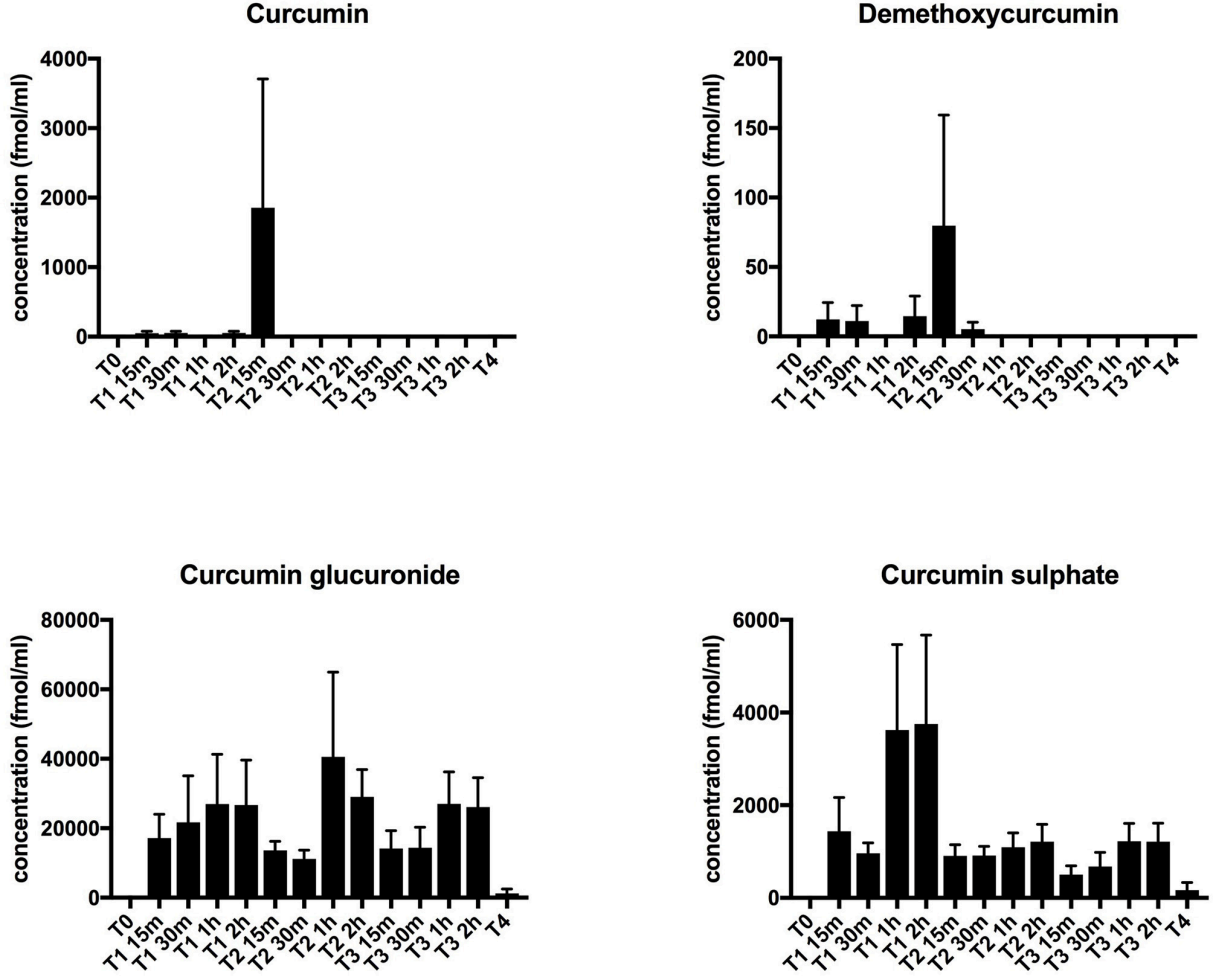
Plasma curcumin levels. Plasma levels of curcuminoids (curcumin and demethoxycurcumin) and their main metabolites (curcumin glucuronide and curcumin sulfate) were determined by LC-ESI-MS/MS. Concentrations are shown in fmol/mL. Results are depicted as mean + standard error of mean. Time points are as follows: T0-baseline, T1-day 1 of treatment, T2-day 7 of treatment, T3-day 14 of treatment, T4, 1 week after last CP dose.

No curcuminoids nor their metabolites, which have also been reported to exert immunomodulatory effects (6, 33), were detectable in plasma at baseline, i.e., before curcumin intake. The two most abundant curcuminoids, curcumin, and demethoxycurcumin in free form, remained undetectable in plasma upon curcumin intake, except for a few outliers. However, its conjugated metabolites, curcumin glucuronide and curcumin sulfate, became detectable after supplementation, with slightly increased levels after 1–2 h (Figure 2). This profile is similar to previous studies and shows that CP uptake was efficient (23, 24). Since curcumin has been shown previously to exert anti-inflammatory effects (6), we performed an extensive interrogation of a broad set of inflammatory mediators at different time points during treatment (at baseline, on the first day of curcumin intake, then once weekly during the 2-week treatment period, and finally 1 week after the end of treatment). In Table 3, we summarize the levels measured at baseline and at the last day of CP supplementation. For a substantial number of analytes however, we noted that values were below the detection limit in > 3 out of 6 patients, so we considered these analytes as undetectable. Furthermore, we noted that absolute values often varied considerably among patients, leading to high standard deviations. No significant changes following CP for any of the tested inflammatory mediators could be noted.

**Table 3:** Soluble inflammatory biomarkers.

**Marker**	**Mean** **±** **SD at time point**		***P*-value**
	**T0**	**T3**	
CA125 (kU/L)	27.29 ± 15.57	27.67 ± 17.35	0.8862
CRP (mg/L)	4.86 ± 5.178	4.55 ± 4.852	0.8548
Neopterin (nmol/L)	14.55 ± 7.552	11.25 ± 5.535	0.1563
Lactate (nmol/μl)	75.81 ± 29.04	101.2 ± 24.89	0.3125
HMGB1 (ng/ml)	1.437 ± 0.473	1.966 ± 0.7599	0.3125
PGE_2_ (pg/ml)	9367 ± 5982	6534 ± 4137	0.3125
CA15-3 (pg/ml)	29163 ± 12464	26622 ± 14116	0.5625
MIF (pg/ml)	233768 ± 569219	1495 ± 994.4	0.8438
Leptin (pg/ml)	35740 ± 20517	33229 ± 21924	0.2188
CEA (pg/ml)	10767 ± 10642	7655 ± 6203	0.3125
Prolactin (pg/ml)	13342 ± 4489	51089 ± 90081	1.0000
BDNF (pg/ml)	2611 ± 754.7	4506 ± 1914	0.0625
EGF (pg/ml)	57.44 ± 33.38	97.34 ± 58.7	0.2188
Eotaxin (CCL11) (pg/ml)	127.9 ± 43.43	125.4 ± 45.2	0.6875
FGF-2 (FGF basic) (pg/ml)	225.4 ± 311	275.2 ± 271.9	0.5625
GM-CSF (pg/ml)	Undetectable	Undetectable	N/A
GROα (CXCL1) (pg/ml)	97.76 ± 121.3	96.44 ± 107.5	0.5625
HGF (pg/ml)	790.6 ± 348	862.6 ± 374.3	1.0000
IFNγ (pg/ml)	68.72 ± 33.59	56.03 ± 29.75	0.0625
IFNα (pg/ml)	Undetectable	Undetectable	N/A
IL-1RA (pg/ml)	Undetectable	Undetectable	N/A
IL-1β (pg/ml)	1.997 ± 1.534	2.244 ± 1.655	0.3125
IL-1α (pg/ml)	Undetectable	Undetectable	N/A
IL-2 (pg/ml)	Undetectable	Undetectable	N/A
IL-4 (pg/ml)	Undetectable	Undetectable	N/A
IL-5 (pg/ml)	Undetectable	Undetectable	N/A
IL-6 (pg/ml)	23.7 ± 13.81	31.2 ± 18.75	0.6466
IL-7 (pg/ml)	Undetectable	Undetectable	N/A
IL-8/CXCL8 (pg/ml)	Undetectable	Undetectable	N/A
IL-9 (pg/ml)	Undetectable	Undetectable	N/A
IL-10 (pg/ml)	Undetectable	Undetectable	N/A
IL-12p70 (pg/ml)	2.389 ± 0.4030	2.428 ± 0.4384	0.8438
IL-13 (pg/ml)	3.79 ± 2.26	4.375 ± 2.243	0.6250
IL-15 (pg/ml)	Undetectable	Undetectable	N/A
IL-17A (pg/ml)	Undetectable	Undetectable	N/A
IL-18 (pg/ml)	151.2 ± 123.1	134.9 ± 65.02	0.6875
IL-21 (pg/ml)	Undetectable	Undetectable	N/A
IL-22 (pg/ml)	Undetectable	Undetectable	N/A
IL-23 (pg/ml)	Undetectable	Undetectable	N/A
IL-27 (pg/ml)	290.1 ± 474.4	206.4 ± 381	0.4606
IL-31 (pg/ml)	Undetectable	Undetectable	N/A
IP-10 (CXCL10) (pg/ml)	101.1 ± 24.93	94.2 ± 15.44	0.2188
LIF (pg/ml)	28.28 ± 55.95	28.16 ± 50.19	0.8438
MCP-1/CCL2 (pg/ml)	58.96 ± 28	81.36 ± 22.73	0.1563
MIP-1α/CCL3 (pg/ml)	61.12 ± 78.37	58.01 ± 67.72	1.0000
MIP-1β/CCL4 (pg/ml)	282.6 ± 179.7	273.4 ± 156.7	0.6875
βNGF (pg/ml)	93.28 ± 120	104.5 ± 100	0.3125
PDGF-BB (pg/ml)	192.3 ± 102.9	243.9 ± 150.6	0.3125
PIGF-1 (pg/ml)	210.8 ± 195.5	272 ± 185.8	0.4375
RANTES/CCL5 (pg/ml)	240 ± 31.88	248.1 ± 72	0.8434
SCF (pg/ml)	19.19 ± 25.56	19.7 ± 23.24	0.5625
SDF1α/CXCL12 (pg/ml)	1372 ± 585.3	1349 ± 448.3	1.0000
TNFα (pg/ml)	Undetectable	Undetectable	N/A
TNFβ/LTA (pg/ml)	Undetectable	Undetectable	N/A
VEGF-A (pg/ml)	999.6 ± 1109	1129 ± 994.7	0.5625
VEGF-D (pg/ml)	Undetectable	Undetectable	N/A

*This table shows the plasma levels of inflammatory biomarkers as measured by ELISA or luminex. Units are mentioned for each marker. Values are presented as mean ± standard deviations. P-values were calculated using the nonparametric Wilcoxon matched-pairs test with Prism software. Analytes were considered undetectable if values were below detection limit in >50% of samples. T0 – baseline, T3 – day 14 of treatment*.

### COX-2 Expression in Immune Cells

**Figure 3:**
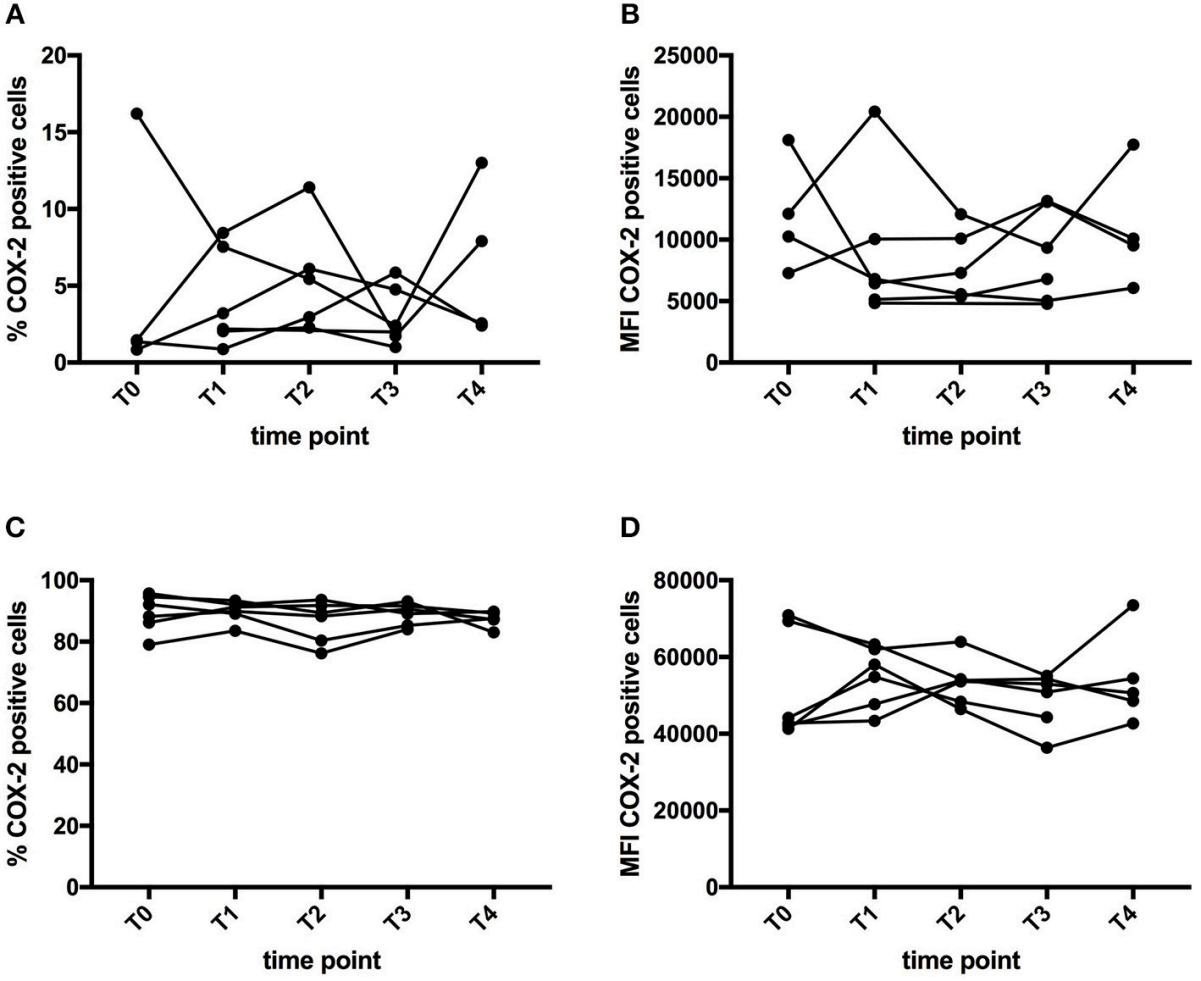
Effect of curcumin supplementation on COX-2 expression. COX-2 expression in PBMC was determined by flow cytometry. The graphs show the expression in the monocyte (CD14^+^) gate, either expressed as percentage of cells expressing COX-2 **(A,C)** or as MFI of COX-2 expression levels **(B,D)**. **(A,B)** Show the expression of COX-2 in PBMC directly after thawing, while **(C,D)** show COX-expression levels after a 5h-*in vitro* culture period in the presence of LPS. Each line depicts one patient. Time points are as follows: T0-baseline, T1-day 1 of treatment, T2-day 7 of treatment, T3-day 14 of treatment, T4, 1 week after last CP dose.

COX-2 is a well-known target of curcumin (34, 35). COX-2 expression was examined by flow cytometry in monocytes, B cells, NK cells, and T cells. Both the percentage and mean fluorescence intensity (MFI) of COX-2 expression in each cell type was measured, both on freshly thawed PBMC as well as on PBMC cultured *in vitro* in the presence of LPS. As expected, COX-2 expression in freshly thawed PBMC was very low, with highest percentage in monocytes. After LPS stimulation, COX-2 expression was increased, mainly in monocytes. Figure 3, shows the percentage of COX-2 expressing cells and the MFI in the monocyte population of freshly thawed PBMC and LPS-stimulated PBMC. We were unable to demonstrate significant changes in COX-2 expression upon CP supplementation (Figure 3). The same analysis was performed for COX-2 expression in B cells, NK cells and T cells, without significant differences (data not shown).

### Immune Cell Subsets

Evidence suggests the capability of curcumin to modulate the frequency and cellular response of different cell types of the immune system during cancer (36–38). In whole blood, we verified the effect of curcumin on the Neutrophil-to-Lymphocyte Ratio (NLR) and the frequency of Myeloid-Derived Suppressor Cells (MDSC) and their expression level of Arginase-1. Three MDSC subtypes were analyzed: CD15^+^ granulocytic, CD33^+^ granulocytic, and CD33^+^ monocytic MDSC. Finally, we assessed the Arginase-1 expression level for these 3 different MDSC subtypes. No significant differences were observed after CP supplementation, neither for NLR nor for MDSC frequencies or their Arginase-1 levels (data not shown).

**Figure 4:**
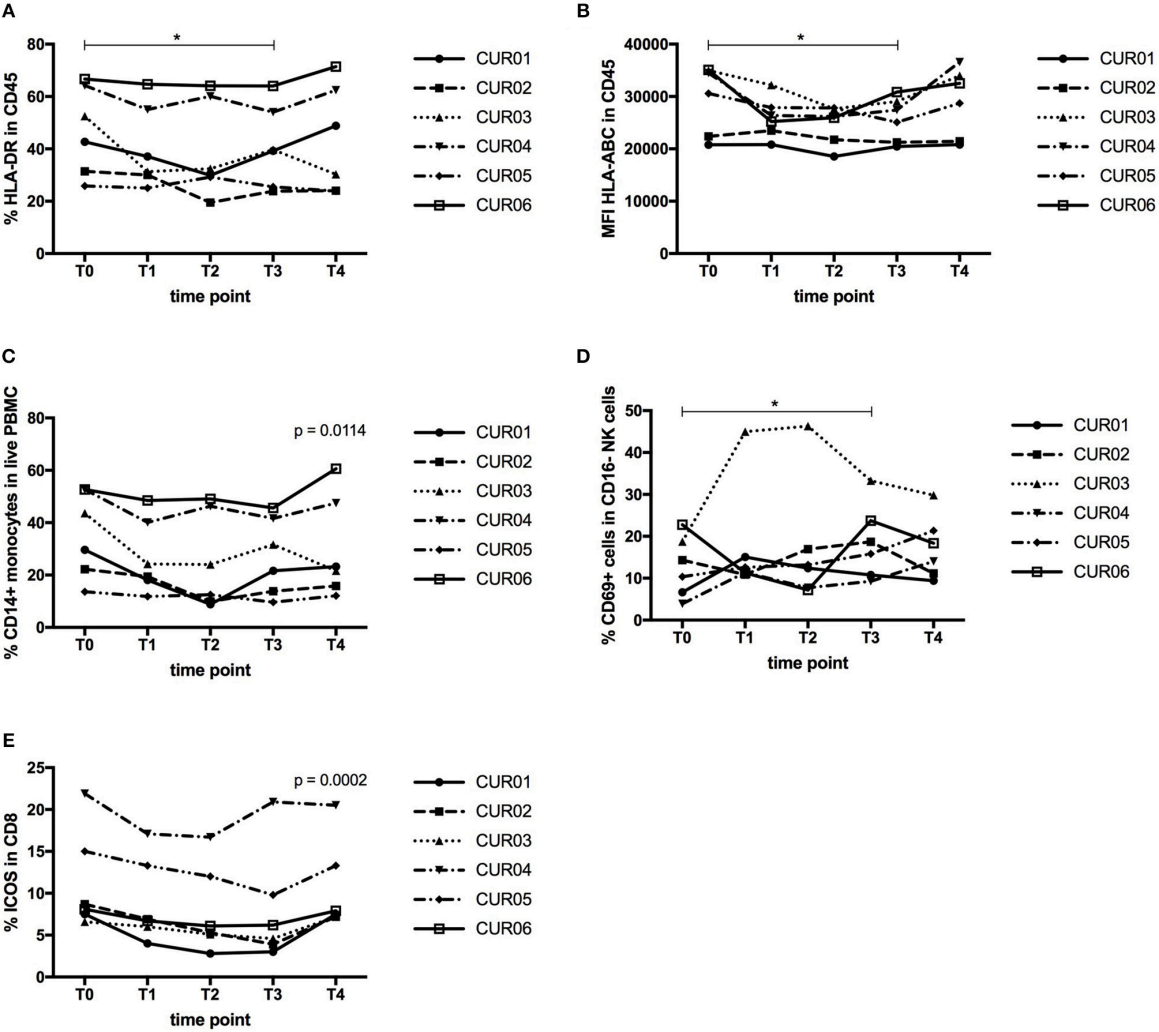
Effect of curcumin supplementation on immunological cell types. **(A)** Expression of HLA-DR by CD45^+^ leukocytes determined by flow cytometry, expressed as percentage of HLA-DR expressing CD45^+^ leukocytes. **(B)** Expression of HLA-ABC by CD45^+^ leukocytes determined by flow cytometry, shown as mean fluorescence intensity (MFI) of HLA-ABC expressed by CD45^+^ leukocytes. **(C)** Percentage of CD14^+^ monocytes in the total PBMC population determined by flow cytometry. **(D)** Percentage of CD69-expressing cells within the CD56^+^ CD16^−^ NK cell population measured by flow cytometry. **(E)** Percentage of ICOS-expressing cells within the CD3^+^ CD4^−^ CD8^+^ T cell population measured by flow cytometry. Each line depicts one patient. Time points are as follows: T0, baseline, T1-day 1 of treatment, T2-day 7 of treatment, T3-day 14 of treatment, T4, 1 week after last CP dose. *P* values were determined using the Wilcoxon signed rank test **(A,B,D)** or the one-way repeated measure analysis of variance (ANOVA) test **(C,E)** with Prism software. ^*^P < 0.05.

We then isolated PBMC and investigated the total leukocyte population and their expression of MHC molecules. As shown in Table 4 and Figures 4A,B, despite a constant total percentage of leukocytes, we observed a significant decline in the frequency of HLA-DR expressing leukocytes and a significant reduction in the expression level of HLA-ABC upon CP treatment (*P* < 0.05). This effect was transient and levels were restored 1 week after discontinuation of curcumin intake.

**Table 4:** Effect of curcumin supplementation on total leukocytes.

**Cell type**	**Mean** **±** **SD at time point**		***P*-value**
	**T0**	**T3**	
% CD45+ cells	99.47 ± 0.3502	97.9 ± 3.659	0.4099
% HLA-ABC by CD45+	99.97 ± 0.05164	99.92 ± 0.2041	1.000
% HLA-DR by CD45+	47.22 ± 16.88	41.03 ± 15.74	0.0313
% HLA-E by CD45+	4.85 ± 6.392	3.467 ± 5.411	0.625
% HLA-G by CD45+	5.967 ± 4.978	4.9 ± 4.626	0.4375
MFI HLA-ABC by CD45+	29719 ± 6539	25681 ± 4199	0.0313
MFI HLA-DR by CD45+	6751 ± 2778	5759 ± 1745	0.0625
MFI HLA-E by CD45+	8008 ± 754.3	7741 ± 635.4	0.4375
MFI HLA-G by CD45+	5759 ± 3365	6658 ± 4823	0.4375

*This table depicts the results of the flow cytometric analysis of total leukocytes (CD45^+^ cells) and their expression of HLA molecules (expressed both as percentage and MFI). Data are expressed as mean ± standard deviations. P-values were calculated using the nonparametric Wilcoxon matched-pairs test with Prism software. T0, baseline; T3, day 14 of treatment*.

Next, we analyzed whether CP intake exerted effects on the innate immune cell types (monocytes, dendritic cells, NK cells). The percentage of CD14^+^ monocytes declined over time upon CP treatment, but their expression levels of MHC molecules remained unaltered (Figure 4C). Another important innate immune cell type is the DC, consisting of two major subsets, myeloid DC (mDC) and plasmacytoid DC (pDC). We also assessed the expression of HLA-ABC, HLA-DR, and CD54, as a measure of their functionality. However, we did not find any changes in their frequency or expression of HLA-ABC, HLA-DR, or CD54 (data not shown). Natural killer (NK) cells constitute an important line of defense in the immune system. CD16^+^ NK cells are considered as the cytotoxic subset of NK cells, while CD16^−^ NK cells are classified as the cytokine-producing NK cell subset. On both cell types, we assessed the expression of the activation markers CD161, CD69, and HLA-DR. CP supplementation did not result in significant changes in either NK cell population (data not shown). However, the percentage of CD69 expressing CD16^−^ NK cells increased significantly upon treatment (Figure 4D, *P* < 0.05).

Finally, we assessed the effects of curcumin on the T lymphocyte compartment. T lymphocytes play a prominent role in tumor immunology, because of the capacity of cytotoxic CD8^+^ T cells to kill tumor cells or the ability of Treg to suppress tumor-specific immunity. Neither on the general T cell subsets, CD3^+^ T cells, CD4^+^ T cells or CD8^+^ T cells, nor on Treg (CD3^+^ CD4^+^ CD8^−^CD25^+^ FoxP3^+^ CD127^lo^), could we observe changes following CP supplementation (data not shown). Next, we assessed the expression of the activation markers CD69, CD137, HLA-DR, ICOS, CTLA-4, PD-1 and Tim-3 on CD4^+^ and CD8^+^ T cells and we observed a significant decline in ICOS expression by CD8^+^ T cells after CP supplementation (Figure 4E). For CD4^+^ T cells, this marker also declined but not significantly. The other activation markers remained unaltered (data not shown). We also investigated the effect of curcumin on the composition of the memory T cell repertoire, but found no significant differences. The T cell antigen receptor (TCR) zeta (TCRζ) chain is an essential component of the TCR complex. Loss of TCRζ is frequently observed in cancer and indicates immunosuppression by MDSC (39). TCRζ expression was measured as TCRζ MFI index (40) in CD3^+^, CD4^+^, CD8^+^ T cells, as well as in CD56^+^ and CD16^+^ NK cells, but we did not find changes upon CP treatment (data not shown).

### Quality of Life Scores

Complete EORTC QLQ-C30 and EQ-5D scores from baseline and the last day of curcumin intake were available for 5 out of 7 patients. One patient did not complete the EORTC QLQ-C30 questionnaire on the last day of curcumin intake and one patient partially completed the EQ-5D questionnaire on the last day of curcumin intake. Changes in QoL scores upon CP supplementation are shown in Table 5. No significant changes in QoL could be noted.

**Table 5:** Quality of life scores.

**Scale**	**Baseline**	**Treated**	***P*-value**
**EORTC QLQ-C30 questionnaire**			
Summary score	80.67 ± 15.52	93.14 ± 4.179	0.1250
Physical functioning	82.22 ± 12.41	84 ± 15.35	>0.9999
Role functioning	83.33 ± 21.08	93.33 ± 14.91	>0.9999
Emotional functioning	61.11 ± 20.86	70 ± 24.01	>0.9999
Cognitive functioning	83.33 ± 21.08	90 ± 9.131	>0.9999
Social functioning	80.56 ± 16.38	93.33 ± 14.91	0.5000
Global QoL	62.5 ± 20.24	79.17 ± 8.33	0.2500
Fatigue	27.78 ± 18.26	13.33 ± 14.49	0.2500
Nausea and vomiting	19.44 ± 34.02	3.334 ± 7.455	>0.9999
Pain	16.67 ± 16.67	13.33 ± 13.94	0.5000
Dyspnoea	11.11 ± 17.21	6.666 ± 14.91	N/A
Insomnia	44.45 ± 40.37	26.67 ± 27.89	>0.9999
Appetite loss	27.78 ± 32.77	0 ± 0	>0.9999
Constipation	11.11 ± 17.21	0 ± 0	0.5000
Diarrhea	11.11 ± 17.21	6.666 ± 14.91	>0.9999
Financial	5.555 ± 13.61	6.666 ± 14.91	N/A
**EQ-5D Questionnaire**			
EQ-index	0.7283 ± 0.1472	0.715 ± 0.2161	0.8750
EQ-VAS	69.17 ± 11.77	79.8 ± 6.419	0.1250

*This table shows the QoL scores from the QLQ-C30 and the EQ-5D questionnaires calculated with SPSS software. QoL scores are presented as means ± standard deviations. P-values were calculated using the nonparametric Wilcoxon matched-pairs signed rank test with Prism software*.

## Discussion

In summary, although the CP formulation was taken up in the blood of the patients, we only detected minor immunological effects. We observed a downregulation of MHC expression by leukocytes, a reduction in the frequency of monocytes and a decreased ICOS expression by CD8^+^ T cells upon CP intake, while the level of CD69 on CD16- NK cells was upregulated. We did not find significant changes in inflammatory biomarker levels, frequencies of other immune cell types, T cell activation and COX-2 expression. A non-significant trend to improved QoL was observed.

A major shortcoming of our study is the small population size and a high inter-patient variability, which might mask small effect sizes. Furthermore, the supplementation period was only 2 weeks, which might be too short to reveal small changes.

Several studies have observed changes in inflammatory biomarkers upon curcumin intake. The absence of changes in inflammatory biomarker levels seems contradictory to other studies where CP treatment has shown to decrease the levels of several inflammatory markers such as CRP (25), IL-1β (41), IL-6 (8, 41, 42), IL-22 (43), sCD40L (41). However, in our study, the levels of a substantial number of analytes were below the detection limit, so further decreases cannot be detected. Together with the above-mentioned small population size and high variability, this might explain this discrepancy.

We were unable to demonstrate significant changes in COX-2 expression upon CP supplementation, which is in contrast to findings in pancreatic cancer (34). Despite the higher dosing of the curcumin complex used by Dhillon et al., the plasma concentrations of curcumin metabolites upon curcumin intake were similar to the levels observed in our study. Moreover, COX-2 reduction by Dhillon et al was measured after only 8 days of supplementation, while we measured COX-2 expression on the first day of intake, after 1 week and after 2 weeks and did not observe a reduction at any of the time points. Both studies however, use a different method to assess COX-2 expression, which could have a different sensitivity. This might explain the higher percentage of COX-2 positivity in PBMC observed by Dhillon et al. compared to our results. However, basal COX-2 expression levels in PBMC reported previously in literature are in general more comparable to the levels observed in this study and it has been shown that LPS stimulation is needed to increase COX-2 expression (44, 45).

We observed a significant decline in the frequency of HLA-DR expressing leukocytes and a significant reduction in the expression level of HLA-ABC upon CP treatment. It has been described previously that curcumin can downregulate MHC class II gene expression by inhibiting IFNγ signaling (46). This might also be the explanation for our results, although we only observed a trend toward decreased IFNγ content in patient plasma upon curcumin intake when comparing the baseline with the end of treatment value (*P* = 0.0625, Wilcoxon matched-pairs test; Table 2). However, the repeated measures ANOVA that compares the effect of curcumin at all time points did indicate a significant decrease of IFNγ concentration in plasma upon curcumin intake (*P* = 0.0189, data not shown). Since we did not have tumor tissue available in this study, we could not investigate whether the downregulation of HLA molecules is also measurable in tumor cells.

Very little evidence about the effect of curcumin on immunological cell types is available. Our data about the absence of effect of curcumin on MDSC frequencies in blood are in contrast with a report in the 4T1 mouse model where curcumin showed a trend toward MDSC reduction in blood (predominantly granulocytic MDSC), which became significant when curcumin was combined with a listeria vaccine (42). A significant decrease in the percentage of monocytes upon curcumin treatment was also observed in an asthma model (47). Our results on activation of NK cells are in agreement with a recent report on the effects of a nanocurcumin formulation, wherein increased NK cell activity was found (48). However, curcumin has also been reported to increase the frequency of NK cells in clinical studies and animal models (49), which we did not observe. Previous data in the literature show that curcumin can increase CD4^+^ and CD8^+^ T cells but also Treg, which we could not observe (49). We did not observe changes in the memory T cell repertoire upon CP intake, while another study showed that curcumin could restore central memory T cell (T_CM_) and effector memory T cell (T_EM_) populations in tumor-bearing mice, but they compared untreated and curcumin-treated tumor-bearing mice and did not assess the effect of curcumin treatment in the same mouse (50).

We observed a significant decline in ICOS expression by CD8^+^ T cells after CP supplementation (Figure 4E). ICOS is a co-stimulatory molecule of the CD28-B7 superfamily and its role in cancer is controversial. On the one hand, data support a role of ICOS:ICOSL in facilitating the anti-tumor T cell response because of observations that diminished ICOS levels in blood associate with worse prognosis in colon cancer and that high ICOS expression on tumor-infiltrating lymphocytes in metastatic melanoma lesions was associated with better post-recurrence survival. On the other hand, an inhibitory, pro-tumor role has been attributed to ICOS signaling related to its function in Treg homeostasis, thus facilitating tumor immune evasion (51). Data also indicate that the ICOS:ICOSL pathway is required for optimal antitumor responses mediated by anti-CTLA-4 therapy (52). The available data about the effect of curcumin on ICOS in literature are scarce and contradictory and mainly come from *in vitro* systems using supra-physiological curcumin concentrations (53, 54).

The absence of changes in QoL scores indicates the absence of toxicities related to curcumin intake, which has also been shown in other studies (1, 55). However, in a randomized controlled trial, curcuminoid supplementation was associated with a significant improvement in QoL compared to placebo (56). We also observed a trend to increased QoL scores upon CP supplementation with both questionnaires used, although not significant. Since the CP dose was tolerable, increasing the dosage of CP could also be considered in further studies. Lack of significance might be explained by the small number of patients in our study or by the short period of supplementation or by the fact that in the paper of Panahi et al the pre-treatment QoL score for the curcuminoid group was lower compared to the placebo group while the post-treatment scores were equal, which might indicate a randomization problem for the QoL parameter.

In conclusion, we observed only minor immunomodulatory effects of curcumin supplementation in endometrial cancer patients. The QoL scores confirmed the absence of toxic effects by curcumin supplementation, but no improvement in QoL is seen. It remains to be explored whether different supplementation regimens or schemes could induce immunological benefit in endometrial cancer.

## Ethics Statement

This study was carried out in accordance with the recommendations of Ethische Commissie Onderzoek UZ Leuven with written informed consent from all subjects. All subjects gave written informed consent in accordance with the Declaration of Helsinki. The protocol was approved by the Ethische Commissie Onderzoek UZ Leuven.

## Author Contributions

ST conceived the project, designed research, performed experiments, interpreted data, and wrote the paper. KR designed research and edited the paper. TE performed the experiments. AVN interpreted data and edited the paper. FA conceived the project and edited the paper.

### Conflict of Interest Statement

The authors declare that the research was conducted in the absence of any commercial or financial relationships that could be construed as a potential conflict of interest.
